# Barriers and competencies in nursing care for diabetic foot management: a mixed-methods observational study

**DOI:** 10.1017/S1463423625100698

**Published:** 2025-12-29

**Authors:** Mónica Rodríguez Valiente, Roberto Carlos Martínez Alcaraz, Javier Sánchez-Gálvez, Francisco Mateo Ramírez, Juan Jesús Baño Egea, María Cristina Sole-Augustí, Arturo Pereda Más, María Dolores Beteta Fernández

**Affiliations:** 1 Virgen de la Arrixaca University Clinical Hospital – IMIB, UDICA – Teaching, Research and Healthcare Quality Unit, Murcia, Spain; 2 Faculty of Nursing, UCAM Universidad Católica de Murcia, Murcia, Spain; 3 Research Group on Molecular and Cellular Biology Solutions in Regenerative Medicine, UCAM, Murcia, Spain; 4 Biomedical Research Institute of Murcia Pascual Parrilla–IMIB, Murcia, Spain; 5 Aljucer Health Centre, Diabetic Foot Unit, Area I, Murcian Health Service, Spain; 6 International University of La Rioja, Spain; 7 Professional Development Unit, Directorate-General of Human Resources, Murcian Health Service, Spain; 8 Directorate of Nursing for Continuity and Care, Area I, Murcian Health Service, Spain

**Keywords:** Attitudes, continuing, diabetic foot, education, health knowledge, practice, Primary Care nursing, professional autonomy

## Abstract

**Aim:**

To explore nurses’ perceptions regarding their knowledge, degree of autonomy, and the difficulties encountered in managing diabetic foot in Primary Care.

**Background:**

Diabetes mellitus is a chronic condition with a high prevalence in Spain, predominantly type 2. One of its most serious complications is diabetic foot disease, affecting between 19% and 34% of patients and associated with considerable morbidity and amputation risk. Primary Care, particularly nursing professionals, plays a pivotal role in the prevention, assessment, and management of diabetic foot. However, institutional, methodological, and personal barriers continue to affect care quality.

**Methods:**

A descriptive, cross-sectional observational study was conducted using quantitative and qualitative methods. A validated ad hoc questionnaire was administered to 176 nurses from the Murcian Health Service participating in a blended learning course on diabetic foot. Variables assessed included professional autonomy, knowledge, dressings use, clinical documentation, training, and perceived challenges. Qualitative analysis was based on open-ended responses using content analysis.

**Findings:**

A total of 88.1% of nurses reported autonomy in performing foot examinations; however, only 45.5% managed wound care independently. Just 19.9% considered themselves sufficiently trained, while 42.6% felt confident in selecting dressings appropriate to the healing phase. Although 56.8% regularly completed specific clinical documentation forms, many still expressed uncertainty about dressing use. Qualitative analysis identified five key barriers: lack of knowledge, patient complexity, institutional constraints, issues of authority and communication, and professional insecurity. These findings provide a current picture of persistent barriers in diabetic foot care and reinforce the need for targeted training and institutional support.

## Background

Diabetes mellitus (DM) is a metabolic disorder characterized by chronic hyperglycaemia, accompanied to varying degrees by disturbances in the metabolism of carbohydrates, proteins, and lipids. When sustained over time, this condition can cause serious damage to target organs such as the heart, eyes, kidneys, blood vessels, and nerves. The most widely used classification of DM is that of the American Diabetes Association, which is based primarily on aetiology and pathophysiological features, and includes four categories: type 1 diabetes, type 2 diabetes, gestational diabetes, and other specific types of diabetes (Elliott and Pfotenhauer, 2022). In Spain, the prevalence of DM based on data from Primary Care clinical records is estimated at 6.8%, equivalent to approximately 3.07 million diagnosed individuals, with type 2 diabetes accounting for 96.6% of cases and type 1 for 3.4% (Ministry of Health, 2020).

According to the national consensus document on strategies to improve the prevention and management of diabetic foot, this condition represents a serious complication affecting between 3% and 4% of people with diabetes. The lifetime risk of developing an ulcer related to this condition ranges between 19% and 34%. Around 70% of these ulcers remain unhealed after 20 weeks of treatment, and their prognosis is significantly worsened by the presence of ischaemia or infection. Peripheral arterial disease is estimated to be present in over 50% of patients affected, particularly in middle- and high-income countries. Moreover, infection occurs in nearly 60% of cases and is the leading cause of lower limb amputation (Lázaro Martínez *et al.*, 2021).

The same consensus highlights that risk stratification and preventive measures are responsibilities shared by Primary Care physicians and nurses. These professionals are tasked with assessing patients’ risk of ulceration through appropriate anamnesis, neuropathy and vascular assessment, and the identification of foot deformities. When deemed necessary, they are also responsible for referring patients to specialist care.

Within the framework of the Best Practice Spotlight Organisations programme, and in alignment with the implementation of nursing best practices, it is essential to identify the barriers encountered by nurses in managing patients with diabetic foot disease in order to optimize care delivery (Belmar *et al.*, 2018).

Several methodological, institutional, and personal barriers can be traced back to undergraduate training and persist into professional practice, creating gaps and obstacles to the application of evidence-based knowledge. This situation has a direct impact on the quality of patient care. In the field of wound care specifically, practices based on inadequately transmitted knowledge – rooted in personal beliefs, habits or traditions lacking scientific support – still prevail. These practices are found both among clinical and academic nurses, are passed on to new generations of professionals, and contribute to variability in the management of patients with wounds (Sánchez-Gálvez *et al.*, 2024).

The aim of this study was to explore nurses’ perceptions regarding their knowledge, autonomy, and the difficulties they face in the management of patients with diabetic foot in Primary Care.

## Methods

### Study design

A descriptive, cross-sectional observational study was conducted, employing a quantitative methodology complemented by qualitative analysis. The aim was to examine the practices, competencies, and perceived barriers among Primary Care nurses within the Murcian Health Service (Servicio Murciano de Salud, SMS) in the management of diabetic foot, including the treatment of active ulcers. The study was based on a structured, ad hoc questionnaire designed and validated by experts in the field, panel of five professionals: two nurses specialized in diabetic foot management, one podiatrist with more than ten years of clinical experience in wound care, one nursing academic with a PhD in clinical research, and one senior nurse educator certified in chronic wound management. The instrument included both closed-ended questions (Likert-type, dichotomous, and multiple choice) and a single open-ended question to delve into perceived challenges. The STROBE Statement (Strengthening the Reporting of Observational Studies in Epidemiology) was used as the reference method (von Elm *et al.*, 2007).

The study was embedded in a blended-learning training course promoted by the SMS Professional Development Unit and the Teaching, Research and Care Quality Unit (UDICA) in Health Area 1. The course was aimed at nursing professionals and family and community nursing residents across all SMS health areas. The online component, hosted on the IDEA platform, included theoretical content, assessment activities, and educational dynamics such as an interactive ‘The Alphabet Game’ – style game. The questionnaire was administered at the beginning of the course as part of a diagnostic activity prior to the training phase.

The in-person component of the course included hands-on training in vascular assessment (pulse palpation, ankle-brachial Index), neurological assessment (monofilament and tuning fork), visual foot inspection, referral criteria, and clinical case resolution involving active ulcers. These activities allowed for practical evaluation of acquired competencies.

### Study population and sample

The target population consisted of practising nurses working in Primary Care centres across the SMS, totalling an estimated 963 professionals (Ministry of Health, 2023). A non-probabilistic convenience sampling approach was used, recruiting 176 nurses who voluntarily completed the questionnaire. This sample represented approximately 18% of the target population. Although it did not reach the ideal sample size for a 5% margin of error, it was deemed sufficient for identifying relevant trends and conducting a preliminary exploration of perceived barriers and competencies in diabetic foot management.

### Data collection instrument

The ad hoc questionnaire was developed by a group of experts in nursing and diabetic foot care. The instrument (Annex 1) consisted of six closed-ended questions distributed across the following thematic domains:

- Professional autonomy: perceived level of autonomy in the examination and treatment of diabetic foot;
- Knowledge and use of dressings: appropriateness of dressing use according to the wound healing phase and familiarity with materials;
- Clinical documentation: availability and use of documentation and follow-up tools;
- Challenges in clinical management: diagnosis, assessment, and local treatment of ulcers;
- Training and continuing education: self-perceived training level in this area;
- Open-ended question: ‘What other difficulties do you encounter when caring for patients with diabetic foot ulcers?’

The only demographic information collected concerned the health area in which the participant worked; no data were recorded regarding age or sex.

### Data collection

Data were collected between May 2023 and December 2024 via the IDEA training platform of the Murcian Health Service. Participants were recruited through voluntary enrolment in the blended learning course on diabetic foot care, announced via institutional e-mail and the Murcia Salud intranet. The course was open to all Primary Care nurses within the Murcian Health Service. Participation in the questionnaire was voluntary but encouraged as part of the diagnostic phase prior to course certification. No financial or academic incentives were provided. The questionnaire was integrated into the blended-learning course on diabetic foot care. It was presented at the start of the course and completed by participants voluntarily, anonymously, and after providing informed consent. Distribution and access to the survey took place within the virtual learning environment, ensuring participation from professionals across various SMS health areas.

### Data analysis

Quantitative data were analysed using descriptive statistics, including frequencies, percentages, and measures of central tendency (mean and median). The dependent variables included nurses’ perceived autonomy, knowledge level, and confidence in dressing use. Independent variables were years of professional experience and geographic health area. Given that the data did not follow a normal distribution (Shapiro–Wilk *p* < 0.05) and some groups had small sample sizes, non-parametric tests were applied (Mann–Whitney U and Kruskal–Wallis). Statistical significance was set at *p* < 0.05, but no significant associations were found between autonomy, experience, or health area (*p* > 0.05).

Qualitative responses from the open-ended question were subjected to thematic analysis following Braun and Clarke’s framework (2006). Two independent reviewers coded all open-ended responses manually and discussed discrepancies until consensus was reached. Microsoft Excel was used for data organization and frequency counting, whereas SPSS version 27.0 (SPSS, Chicago, IL, USA) was employed exclusively for quantitative statistical analysis, qualitative data were analysed manually. Thematic categories were developed inductively and grouped into five domains:

- Professional knowledge;
- Patient complexity;
- Authority and decision-making communication;
- Institutional factors;
- Other (insecurity, fear, generalized responses).

### Ethical considerations

The study was approved by the Research Ethics Committee of the Catholic University of San Antonio of Murcia (UCAM) under code CE012508 and adhered to the ethical principles set out in the Declaration of Helsinki. All participants provided informed consent, and the confidentiality and anonymity of the data collected were ensured at all times.

## Results

The analysis of the data collected through the structured questionnaire completed by Primary Care nurses from the Murcian Health Service (SMS) provides an overall view of clinical practice, knowledge levels, and perceived challenges in the management of patients with diabetic foot ulcers. A total of 190 questionnaires were distributed, and 176 were fully completed and included in the final analysis (completion rate 92.6%). No missing data were recorded, as the questionnaire required complete responses to proceed.

### Distribution by health area

**Figure 1.**
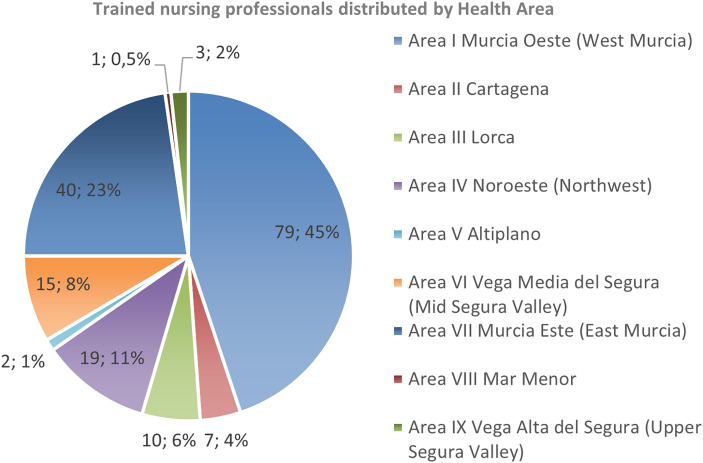
Distribution of trained nursing professionals by Health Area.

Participants were unevenly distributed across the nine Health Areas of the SMS as follows: Area I (Murcia West) 45%, Area VII (Murcia East) 23%, Area IV (Northwest) 11%, Area VI (Vega Media) 8%, and the remaining 13% across other areas. This distribution reflects the population density and service size of each area. (Figure 1).

### Autonomy in clinical practice

A total of 88.1% of professionals reported being autonomous in performing diabetic foot examinations according to their own clinical judgement, while 11.9% stated that they relied entirely on medical instructions.

Regarding wound care management, 45.5% of nurses reported making independent decisions, 24.4% aimed to maintain consistency with colleagues’ practices, 23.3% worked under a consensual treatment plan with periodic reviews, and 6.8% followed medical instructions exclusively (Table 1). No statistically significant differences were found between years of experience or geographic area and perceived autonomy (Kruskal–Wallis *p* = 0.217).

**Table 1.** Autonomy in clinical practice

Question	*n*	%
In my workplace, I have autonomy and perform foot examinations based on my own clinical judgement:		
– Yes, always	155	88.1%
– No. I perform wound care as indicated by the attending physician	21	11.9%
Regarding the treatment of patients with diabetic foot ulcers:		
– Each nurse manages the wound based on their own clinical judgement and criteria	80	45.5%
– Treatment is agreed upon and re-evaluated periodically	41	23.3%
– An effort is made to maintain consistency in treatment among colleagues	43	24.4%
– Treatment is provided according to the physician’s indications	12	6.8%

### Training and continuing education

Findings on training revealed that only 19.9% of participants considered themselves to have sufficient and up-to-date knowledge regarding diabetic foot management. In contrast, 42% felt inadequately trained, while 38.1% expressed uncertainty on this matter. When comparing self-perceived training with years of experience, no significant differences were observed (Mann–Whitney U, *p* = 0.384), suggesting that perceived knowledge gaps were common across all professional groups.

In terms of knowledge and use of dressings, 42.6% of participants felt confident in selecting appropriate dressings according to the wound healing phase. A similar proportion (45.5%) reported that their autonomy depended on the type of dressing required, and 11.9% stated they did not feel adequately prepared (Table 2).

**Table 2.** Training and continuing education

Question	*n*	%
I believe I have sufficient and up-to-date knowledge about diabetic foot:		
– Yes	35	19.9%
– No	74	42.0%
– I’m not sure	67	38.1%
I am autonomous in selecting dressings because I know which to use depending on the wound healing phase:		
– Yes. always	75	42.6%
– No	21	11.9%
– It depends on the dressing	80	45.5%

### Knowledge of dressing use and documentation

With regard to the use of documentation and follow-up tools (Table 3), 56.8% of nurses reported having access to specific forms for recording the use of dressings and stated they completed them systematically. However, 29.5% reported not having access to such forms, and 13.6% stated they used them only occasionally. When asked about confidence in correct dressing use, 43.2% reported feeling secure, while 48.3% expressed uncertainty. Confidence in dressing use did not vary significantly according to health area or professional experience (Kruskal–Wallis *p* = 0.179).

**Table 3.** Knowledge of dressing use

Question	*n*	%
I have access to specific documentation forms to record the dressings I apply to ulcers, and I complete them:		
– I have them and always complete them	100	56.8%
– I do not have access to such forms	52	29.5%
– I have them but only complete them occasionally	24	13.6%
I believe I use dressings correctly:		
– Yes	76	43.2%
– No	15	8.5%
– I’m not sure	85	48.3%

### Perceived challenges (qualitative analysis)

Thematic analysis of the open-ended responses was conducted manually by two independent reviewers using Excel for coding and frequency counts. Agreement between reviewers was high (Cohen’s *κ* = 0.87), indicating strong inter-rater reliability. Difficulties in managing diabetic foot ulcers revealed five key thematic categories (Table 4). The most frequently cited was ‘Professional knowledge’ (55 mentions, 31.3%), followed by ‘Patient complexity’ (45 mentions, 25.6%), ‘Institutional factors’ (22, 25.6%), ‘Authority and communication’ (18, 20.9%), and a final category ‘Other’ (10, 11.6%), which included vague or individualized responses. This manual thematic analysis allowed for the identification of five domains, corroborated independently by two reviewers to ensure reliability. Representative quotations included: ‘I often feel unsure about which dressing is best for each stage of healing’ and ‘Even when we explain care routines, some patients don’t follow advice due to lack of understanding or family support’.

**Table 4.** Perceived challenges in the management of diabetic foot ulcers

Main category	Most frequent subthemes	Estimated frequency
Professional knowledge	Lack of general knowledge, dressings, diagnosis, clinical assessment	55
Patient complexity	Poor treatment adherence, complex wounds, comorbidities	45
Institutional factors	Lack of resources, materials, protocols, access to specialists	22
Authority and decision-making communication	Discrepancies among professionals, absence of protocols, poor coordination	18
Other	Fear, insecurity, vague or non-responses	10

#### Professional knowledge

Participants highlighted a lack of up-to-date knowledge on dressings, wound diagnosis, care protocols, and clinical assessment skills.

#### Patient complexity

Nurses frequently described patients with poor adherence to treatment, inadequate self-care practices, and comorbidities that hinder favourable ulcer progression (i.e., infections, ischaemia, poorly controlled diabetes). The clinical course was often perceived as slow and unpredictable.

#### Institutional factors

Barriers identified included the absence of updated protocols, scarcity of material resources, high workload, and limited access to specialist services. These were considered significant structural limitations to effective diabetic foot management.

#### Authority and communication

Respondents reported discrepancies in decision-making between nurses and physicians, lack of therapeutic consensus, and poor continuity of care between levels of the health system.

#### Other responses

Some responses expressed professional insecurity, fear of causing harm to patients, or provided ill-defined or generalized difficulties. A few participants indicated they encountered no difficulties or responded with general statements such as ‘many’.

## Discussion

The results of this study reveal that, although Primary Care nursing professionals feel relatively autonomous in conducting foot examinations and delivering initial care for patients with diabetic foot, significant gaps remain in specific knowledge and skills required for comprehensive management of this condition. The findings highlight an evident need for ongoing education and training, as reflected in the low proportion (19.9%) of nurses who considered themselves adequately informed and up to date. This training deficit could compromise the quality of care, particularly in key aspects such as appropriate dressing selection and the management of complex ulcers. Supporting evidence from Monteiro-Soares *et al*. (2021) indicates that the quality and accessibility of specialized care are critical to the prevention of amputations, underscoring the importance of strengthening clinical training and promoting evidence-based practice in this field (Monteiro-Soares *et al.*, 2021).

To our knowledge, this is one of the few studies in Spain exploring both the competencies and perceived barriers of Primary Care nurses in diabetic foot management. Our findings complement previous qualitative research on wound care knowledge (Kaya and Karaca, 2018; Chuang *et al*., 2023) and extend understanding of institutional and professional challenges specific to diabetic foot care. In contrast to studies focusing on patients’ experiences (Semerci Çakmak and Özdemir, 2024), our work contributes the professional nursing perspective. Furthermore, Ding *et al*. (2023) highlight the impact of e-learning in improving wound care knowledge, manifesting the importance of continuous professional development in this field.

Although nurses reported a degree of autonomy in treating diabetic foot ulcers, several additional challenges emerged. These included patients’ poor adherence to treatment, a lack of standardized approaches among colleagues, and a general absence of treatment protocols. Respondents placed high value on the presence of experienced professionals in this area and advocated for institutional support through training, standardized clinical guidelines, and the establishment of unified working protocols. Many participants stressed the need for specific units with qualified personnel across different health areas to ensure appropriate treatment and follow-up for these patients. A recurrent theme was the lack of confidence in choosing appropriate treatments for complex diabetic foot cases, likely due to the clinical complexity of such patients and the rapid progression of their condition towards serious complications, such as soft tissue infections or osteomyelitis.

Despite these challenges, nurses expressed a strong interest in gaining further knowledge about managing diabetic foot ulcers and in receiving practical training in the application of advanced wound care techniques. The biological complexity of both the patients and their wounds demands updated educational resources tailored to their needs. Zhao *et al*. (2024) also identified a growing interest in specialized diabetic foot care training, while simultaneously pointing out considerable gaps in the practical preparation of nursing staff – findings consistent with those of this study (Zhao *et al.*, 2024).

Evidence from Zhou and Zhou (2024) further suggests that comprehensive nursing interventions can significantly reduce the rate of lower limb amputations among patients with diabetic foot ulcers. Such interventions not only improve clinical outcomes and quality of life but also help to identify risk factors for amputation, such as age, educational level, and socioeconomic status (Zhou and Zhou, 2024). Addressing these determinants through a multidisciplinary approach – bringing together physicians, nurses, podiatrists, and social workers – is crucial for anticipating clinical deterioration. Likewise, the uncertainty observed in decision-making regarding dressing use and the lack of specific clinical documentation clearly point to the urgent need for standardized protocols and continuous staff training. As highlighted by the literature, creating a supportive organizational and educational environment is essential to enable nurses to carry out their roles with autonomy and competence, thereby optimizing health outcomes for this patient group.

Moreover, the qualitative analysis reveals that the barriers identified are not only individual but also systemic. Organizational issues such as the absence of standardized protocols, lack of resources, and poor interprofessional communication diminish nurses’ autonomy and limit their ability to intervene effectively. These factors contribute to variability in the quality of care provided. The success of efforts to prevent and manage diabetic foot disease depends on a well-organized care team that adopts a holistic approach – viewing the ulcer as a symptom of multisystemic disease – and integrates the various disciplines involved. These findings are in line with the recommendations of the International Working Group on the Diabetic Foot (IWGDF), which emphasize the importance of clear clinical guidelines and collaborative environments for effective diabetic foot management (Schaper *et al.*, 2024).

This study presents several limitations. Firstly, a non-probabilistic convenience sampling method was used, which limits the representativeness of the results and their generalizability beyond the context of the Murcian Health Service. Secondly, the questionnaire was self-administered, which may have introduced social desirability bias, particularly in responses related to professional autonomy or appropriate use of dressings. Additionally, participation was linked to a voluntary training course, which could imply a sample of professionals already motivated by or interested in the topic. Finally, no sociodemographic variables such as age, sex, or years of experience were systematically collected, which restricted the ability to perform more nuanced analyses regarding potential differences in perceived competencies and barriers. Future studies should incorporate them to allow for more nuanced subgroup analyses.

## Conclusions

The findings of this study manifest the importance of implementing targeted training strategies and addressing organizational barriers in order to improve diabetic foot management in Primary Care. Integrating evidence-based care models, alongside clear protocols and institutional support, is essential to fostering the autonomy and confidence of nursing professionals. This, in turn, will contribute to the prevention of severe complications and to the overall improvement of care quality for this vulnerable patient population.

## Supporting information

Rodríguez Valiente et al. supplementary material Rodríguez Valiente et al. supplementary material

## Supplementary material

The supplementary material for this article can be found at https://doi.org/10.1017/S1463423625100698.
