# Supplementary material for: Barriers and competencies in nursing care for diabetic foot management: a mixed-methods observational study
Source: Prim Health Care Res Dev. 2025 Dec 29;27:e3. doi: 10.1017/S1463423625100698 (PMC12780415; doi:10.1017/S1463423625100698)
Supplement: Rodríguez Valiente et al. supplementary material [file S1463423625100698sup001.docx]

**Annex 1.** *Survey for nursing professionals on perceptions and knowledge regarding patients with diabetic foot*

| **PREGUNTAS** | **RESPUESTAS** |
| --- | --- |
| 1. I have autonomy and perform foot examinations based on my own clinical judgement | 1. Yes, always 2. No, I follow the examination as indicated by the attending physician |
| 1. Regarding the treatment of patients with active diabetic foot ulcers | 1. We follow treatment indications based on the physician’s judgement 2. Each nurse manages the wound according to their own knowledge and clinical judgement 3. We try to maintain a consistent approach among nursing professionals 4. Treatment is agreed upon and the patient’s condition is re-evaluated every 15 days or as needed |
| 1. I am autonomous in the use of dressings because I know when each is indicated based on the wound healing phase | 1. Yes, always 2. No 3. It depends, I know how to use some types of dressings |
| 1. I have specific documentation forms to record the dressings applied to ulcers, and I complete them | 1. I have them and always complete them 2. I do not have access to such forms 3. I have them but only complete them occasionally |
| 1. I consider that I have sufficient and up-to-date knowledge about this condition | 1. Yes 2. No 3. I’m not sure |
| 1. I believe I use dressings correctly | 1. Yes 2. No 3. I’m not sure |

**Open-ended question:** *What other difficulties do you encounter when caring for patients with diabetic foot ulcers?*
